# Personalized Medicine in Brain Gliomas: Targeted Therapy, Patient-Derived Tumor Models (Review)

**DOI:** 10.17691/stm2023.15.3.07

**Published:** 2023-05-28

**Authors:** K.S. Yashin, D.V. Yuzhakova, D.A. Sachkova, L.S. Kukhnina, T.M. Kharitonova, A.S. Zolotova, I.A. Medyanik, M.V. Shirmanova

**Affiliations:** Neurosurgeon, Department of Neurosurgery, University Clinic; Privolzhsky Research Medical University, 10/1 Minin and Pozharsky Square, Nizhny Novgorod, 603005, Russia; Assistant, Department of Traumatology and Neurosurgery named after M.V. Kolokoltsev; Privolzhsky Research Medical University, 10/1 Minin and Pozharsky Square, Nizhny Novgorod, 603005, Russia; Oncologist, Polyclinic Department; Nizhny Novgorod Regional Oncologic Dispensary, 11/1 Delovaya St., Nizhny Novgorod, 603126, Russia;; Researcher, Laboratory of Genomics of Adaptive Antitumor Immunity, Research Institute of Experimental Oncology and Biomedical Technologies; Privolzhsky Research Medical University, 10/1 Minin and Pozharsky Square, Nizhny Novgorod, 603005, Russia;; Master Student, Department of Biophysics; National Research Lobachevsky State University of Nizhni Novgorod, 23 Prospekt Gagarina, Nizhny Novgorod, 603950, Russia Laboratory Assistant, Laboratory of Fluorescent Bioimaging, Research Institute of Experimental Oncology and Biomedical Technologies; Privolzhsky Research Medical University, 10/1 Minin and Pozharsky Square, Nizhny Novgorod, 603005, Russia;; Student, Faculty of Medicine; Privolzhsky Research Medical University, 10/1 Minin and Pozharsky Square, Nizhny Novgorod, 603005, Russia;; Student, Faculty of Medicine; Privolzhsky Research Medical University, 10/1 Minin and Pozharsky Square, Nizhny Novgorod, 603005, Russia;; Resident, Department of Neurosurgery, University Clinic; Privolzhsky Research Medical University, 10/1 Minin and Pozharsky Square, Nizhny Novgorod, 603005, Russia;; Neurosurgeon, Department Neurosurgery, University Clinic; Privolzhsky Research Medical University, 10/1 Minin and Pozharsky Square, Nizhny Novgorod, 603005, Russia; Professor, Department of Traumatology and Neurosurgery named after M.V. Kolokoltsev; Privolzhsky Research Medical University, 10/1 Minin and Pozharsky Square, Nizhny Novgorod, 603005, Russia; Oncologist, Polyclinic Department; Nizhny Novgorod Regional Oncologic Dispensary, 11/1 Delovaya St., Nizhny Novgorod, 603126, Russia;; Deputy Director for Science, Research Institute of Experimental Oncology and Biomedical Technologies; Privolzhsky Research Medical University, 10/1 Minin and Pozharsky Square, Nizhny Novgorod, 603005, Russia;

**Keywords:** glioblastoma, astrocytoma, targeted therapy, patient-derived tumor models, organoid, personalized medicine

## Abstract

Gliomas are the most common type of primary malignant brain tumors. The choice of treatments for these tumors was quite limited for many years, and therapy results generally remain still unsatisfactory. Recently, a significant breakthrough in the treatment of many forms of cancer occurred when personalized targeted therapies were introduced which inhibit tumor growth by affecting a specific molecular target. Another trend gaining popularity in oncology is the creation of patient-derived tumor models which can be used for drug screening to select the optimal therapy regimen.

Molecular and genetic mechanisms of brain gliomas growth are considered, consisting of individual components which could potentially be exposed to targeted drugs. The results of the literature review show a higher efficacy of the personalized approach to the treatment of individual patients compared to the use of standard therapies. However, many unresolved issues remain in the area of predicting the effectiveness of a particular drug therapy regimen. The main hopes in solving this issue are set on the use of patient-derived tumor models, which can be used in one-stage testing of a wide range of antitumor drugs.

## Introduction

Gliomas (astrocytomas) are the most common type of primary malignant brain tumors accounting for 80.8% of all malignant brain tumors. According to CBTRUS (Central Brain Tumor Register of the United States), the incidence of primary malignant CNS tumors was 7.06 per 100,000 people from 2014 to 2018 [1]; in Russia, the incidence of malignant brain tumors as of 2021 was 5.64 per 100,000 persons [2]. Among astrocytomas, conventionally benign astrocytoma forms (grade 2) exist characterized by relatively slow growth, and malignant astrocytoma forms (grade 3–4). The most malignant type of astrocytoma is glioblastoma (grade 4).

In spite of the large number of studies conducted in the last 15 years, the current standard of care for primary tumors is quite limited and includes maximum safe resection of the tumor followed by radiotherapy and chemotherapy applying temozolomide. Despite aggressive treatment regimes, the maximum median survival rate is two years [3-5]. Recurrence of tumor growth is inevitable, but no therapy standards exist in this case, and possible treatment options are limited to repeated surgery, the use of bevacizumab anti-angiogenic drug in combination with or without irinotecan, and experimental treatments in clinical trials. Unfortunately, the efficacy of glioblastoma recurrence treatment remains low, the maximum survival rate in this group of patients is 6 months [6].

Continued tumor growth depends on two factors: the impossibility of its total removal and unavailability of highly effective drugs. In terms of tumor biology, two key factors underlie this growth: 1) high heterogeneity within the tumor and significant differences between tumors of different patients [7-10]; 2) high invasiveness and rapid infiltrative growth into the surrounding brain matter [9, 10]. These properties are caused by complex mechanisms of tumor growth involving a large number of different signaling pathways, which determines the high aggressiveness of astrocytomas, their high adaptability, and resistance to most types of therapy.

Methods of molecular genetic study were actively introduced in oncology recently allowing to better understand the biological characteristics of a particular tumor and thus to select the most effective therapy. Selection of targeted drugs specifically affecting the mechanisms regulating a particular tumor growth based on tumor molecular profiling is a new strategy for personalized medicine in gliomas. Another promising area for selection of personalized drug therapy is the creation of patient-derived tumor models.

This paper presents a critical review of current trends of personalized medicine in brain astrocytomas.

To this end, literature was searched reflecting the widespread use of personalized medicine in astrocytomas based on molecular genetic profiling and based on drug screening using tumor models. The PubMed and eLIBRARY.RU databases were used in the course of the work. The scientific papers search depth was from 2004 to 2022 inclusive.

## Current approaches to astrocytomas treatment

Current standards for malignant astrocytomas treatment include maximum safe tumor resection followed by radiation and chemotherapy based on molecular genetic profiling [11, 12]. Gross total tumor resection is often impossible due to infiltrative tumor growth, tumor location near eloquent brain areas which will cause neurological deficit development if damaged [13]. Therefore, tumor recurrence up to 2 cm from the primary tumor occurs in approximately 90–95% of patients with glioblastoma [14, 15]. As a consequence, effective adjuvant therapy is essential for the survival of patients with astrocytomas.

However, the set of drugs for astrocytomas treatment is rather limited and includes temozolomide alkylating agent, different regimens of standard PCV chemotherapy (lomustine, vincristine, procarbazine), irinotecan, etoposide, and bevacizumab as inhibitor of vascular endothelial growth factor. The main drug choice in case of tumor recurrence is bevacizumab in mono-regimen or in combination with irinotecan or lomustine [16].

Currently, the classification of astrocytomas used in clinical practice is largely based on tumor molecular genetic profiling. Routinely defined markers include mutation of isocitrate dehydrogenase (IDH), methylation of O6-methylguanine-DNA methyltransferase (MGMT) promoter, amplification of epidermal growth factor receptor (EGFR), 1p/19q codependency, mutation of telomerase reverse transcriptase (TERT) gene promoter, mutation of *ATRX* gene [13, 17]. Thus, presence of IDH mutation and MGMT promoter methylation is accompanied by increased sensitivity to alkylating drugs and radiation therapy and determines a relatively favorable prognosis for patients with astrocytomas [18]. Accordingly, chemoradiotherapy with temozolomide is less effective in patients with tumors without MGMT promoter methylation [19]. The presence of 1p19q codeletion also determines a relatively favorable prognosis and is a predictor of a good response to standard therapy [20, 21]. On the contrary, the presence of TERT mutation and absence of IDH1/2 mutation is associated with worse survival rates [22].

**Figure 1.**
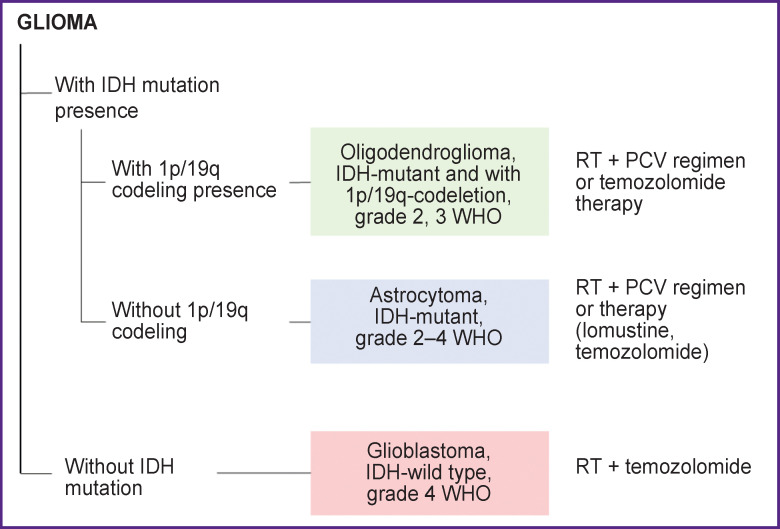
Main diagnostic and prognostic markers of gliomas and the most frequently chosen treatment regimens related to them RT — radiation therapy

Unfortunately, most of the isolated gliomas markers have primarily diagnostic and prognostic value but, unlike many other cancer types, do not allow to determine an effective treatment regimen. The first-line therapy is carried out with temozolomide [23] which showed high efficacy, or with different options of PCV regimen (Figure 1).

## Application of targeted therapy in brain astrocytomas treatment

### Major signaling pathways of regulation and proliferation of tumor cells in astrocytomas

Glioblastoma is characterized by a variety of genetic and epigenetic alterations; however, careful analysis of genetic aberrations in this tumor revealed three main pathogenetic mechanisms of tumor growth: 88% for activation of signaling pathway of tyrosine kinase receptor RTK/RAS/phosphoinositide 3-kinase (PI3K), 87% for inhibition of p53 signaling pathways, and 78% for retinoblastoma protein (Rb) [24, 25]. Currently, the possibility is actively investigated of using targeted drugs to control astrocytoma growth.

RTK activation stimulates PI3K/Akt/PTEN/mTOR signaling pathway, belonging to the main pathways involved in the regulation of cell proliferation, growth, differentiation, metabolism, survival, cell apoptosis, and angiogenesis activation. This pathway ensures rapid preparation of cell protein synthesis mechanism along with rapid tumor growth in response to proliferative stimulus [26, 27]. Activation of this signaling pathway is often associated with aggressive growth and resistance to chemo- and radiation therapy and unfavorable prognosis. In contrast, *TP53* and *Rb* genes are antitumor suppressor genes: p53 protein plays an important role in coordinated cellular stress response by regulating genes involved in apoptosis, DNA repair and neovascularization processes, while hypophosphorylated Rb protein prevents activation of genes responsible for tumor progression through cell cycle activation [26].

It is also worth mentioning RAS/RAF/MAPK kinase pathway found in gliomas, which is a chain of sequentially interacting proteins that transmit signals from the cell surface receptor into the nucleus. The signal transmission controls gene transcription, metabolism, proliferation and motility, cell apoptosis, and angiogenesis. Previously, activating mutations in RAS were considered rare, but a recent study [28] revealed a significant number of KRAS and NRAS mutations in astrocytomas.

**Figure 2.**
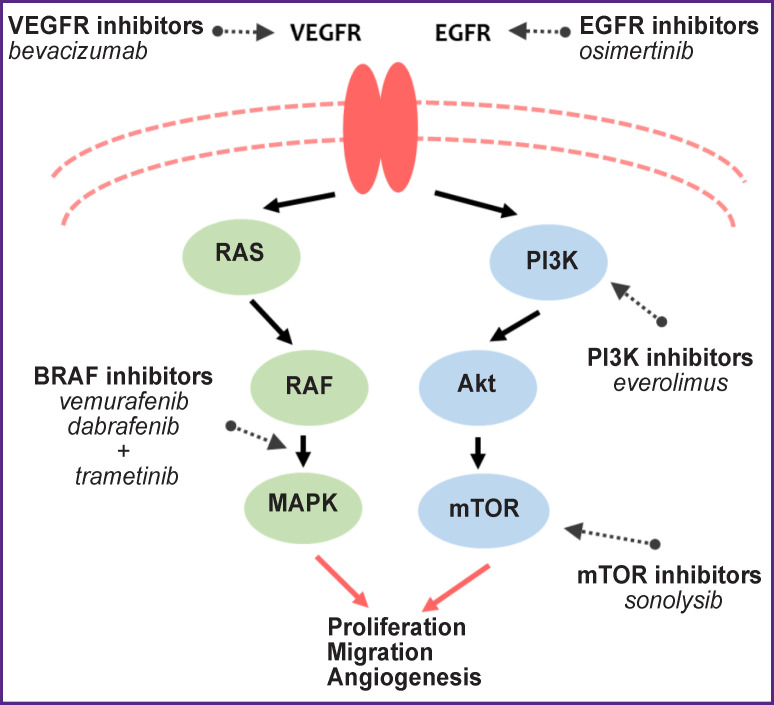
Schematic of RTK/PI3K/Akt/PTEN/ mTOR and RAS/RAF/MAPK signaling pathways that play a key role in glial brain tumor growth with indication of main possible target therapy options

A detailed study of these pathological pathways suggested a number of molecular targets for their inhibition, prevention of tumor growth and proliferation (Figure 2).

### RTK/PI3K/Akt/PTEN/mTOR signaling pathway

#### Inhibitors of epidermal growth factor receptor (EGFR)

 EGFR in glioblastomas plays important role in the RTK/PI3K/Akt/PTEN/mTOR signaling pathway, and its amplification, rearrangement, or point mutations are observed in more than 40% of cases [29]. Given the relatively frequent occurrence, the use of EGFR inhibitors seemed quite promising, especially since these drugs showed excellent results in patients with non-small cell lung cancer. However, in the case of glioblastoma, the first (erlotinib and gefetinib) and second-generation drugs (afatinib, dacomitinib, neratinib) demonstrated no significant effect on survival [30-33]. Possible reasons for ineffectiveness are poor penetration of active molecules through the blood–brain barrier (BBB), high tumor heterogeneity, a variety of possible EGFR mutations, and compensatory opportunities due to other mechanisms of tumor growth.

According to recent data, osimertinib, a third-generation inhibitor used to treat non-small cell lung cancer, including those with brain metastases, has a better ability to penetrate through the BBB [34]. A recent study on xenografts from patients with glioblastoma [35] showed the efficacy of osimertinib in controlling cell growth even with no EGFR expression. This, together with data on ability to effectively overcome the BBB, makes osimertinib a promising drug for treatment. Cases were described of its successful use in patients with malignant astrocytomas [36, 37].

In spite of inconclusive results of studies evaluating the efficacy of EGFR inhibitors in the treatment of high-grade astrocytomas, reports appeared recently on the necessity to revise approaches to the use of this group of drugs based on more profound molecular-genetic tumor typing and selection of a group of patients in whom control of tumor growth can be achieved [38, 39].

#### Inhibitors of vascular endothelial growth factor receptor tyrosine kinase (VEGFR)

 VEGFR is a key regulator of angiogenesis in glioblastoma [40]. Initial studies in patients with recurrent glioblastoma showed efficacy of bevacizumab inhibitor in mono-regimen or in combination with irinotecan, compared to the retrospective group [41, 42]. However, phase III clinical trials for patients with primary glioblastoma demonstrated no higher survival rates in the bevacizumab group compared to controls [43]. Positive clinical and radiological dynamics observed in patients following bevacizumab use are associated with suppression of angiogenesis and reduction of cerebral edema, but this has no sufficient antitumor effect to suppress tumor progression [26]. Other drugs from VEGFR inhibitor group (vatalanib, tivozanib, cediranib, aflibercept, sorafenib) neither showed effectiveness in glioblastoma treatment [26, 44–46].

Further researches are currently underway with drugs that demonstrated relative efficacy in phase II studies (regorafenib, lenvatinib) [47, 48].

A number of studies indicate relative efficacy of apatinib, a low-molecular-weight tyrosine kinase inhibitor, in the treatment of continued growth of glioblastoma targeting VEGFR-2 [49-56].

#### Inhibitors of phosphoinositide 3-kinase (PI3K) and target of mammalian rapamycin (mTOR)

 Mutations of key genes in RTK/PI3K/Akt/PTEN/mTOR signaling pathway are observed in almost 90% of glioblastoma studies [57], this making them one of the most promising objects for targeted therapy. However, phase II studies showed low efficacy of both irreversible PI3K inhibitors (sonolysib [58], buparlysib [59]) and mTOR inhibitors (temsirolimus [60], sirolimus [61], everolimus [62]). A recent phase II study [63] showed that use of paxalisib at the maximum tolerated dose leads to increased time to progression and overall survival in patients with primary glioblastoma.

Everolimus mTOR inhibitor showed no efficacy in patients with primary MGMT-nonmethylated glioblastoma both in monotherapy [64] and in combination with radiotherapy or temozolomide [62]. The authors of another phase II study reported the relative efficacy of adding bevacizumab and everolimus to standard glioblastoma therapy [65]. However, phase III studies are needed to clarify the role of this combination.

### RAS/RAF/MAPK signaling pathway

#### BRAF gene inhibitors

 Activating mutation of *BRAF* V600E gene occurs in 60–80% of pleomorphic xanthroastrocytomas (grade 2–3), in 30% of disembryoblastic neuroepithelial tumors, in 20% of gangliogliomas (grade 1), in 5% of pilocytic astrocytomas (grade 1) [66-68], and also in other astrocytomas [69]. A systematic review [70] demonstrated that the presence of BRAF V600E in astrocytoma is associated with a more favorable prognosis. It was established that testing for the presence of BRAF V600E can be implemented in patients with astrocytomas [71].

Vemurafenib demonstrated good results in patients with highly malignant astrocytomas at BRAF V600E presence [72-74]. Moreover, in glioblastoma with BRAF mutation, examples were described of good response to targeted therapy in case of leptomeningeal spread (usually accompanied by rapid tumor progression) [73].

A study of dabrafenib + trametinib combination in patients with high-grade astrocytoma [75] showed that the response rate was 22% in astrocytoma (grade 3) and 29% in glioblastoma (grade 4). Subsequent studies demonstrated the efficacy of this regimen in patients with relapsed or treatment-resistant astrocytomas in the presence of positive BRAF V600E mutation [71]. Currently, clinical guidelines allow targeted therapy with *BRAF* gene inhibitors by tumor boarddecision if BRAF V600E mutation is present [16].

## Limitations of targeted therapy

Targeted therapy was recognized as a promising trend in oncology due to the attractiveness of the very idea of its targeted effect on tumor growth specific mechanism. Currently, this approach really demonstrated significant efficacy for variety of cancer types. However, no new drugs were proposed to fundamentally improve therapy results in the treatment of brain astrocytomas despite the large number of clinical trials. Moreover, no dependence is often found between the efficacy of various drugs and the presence or absence of a molecular target in the tumor; on the contrary, the relevant drug proves to be effective in cases with no marker in the tumor.

The observed ineffectiveness of targeted drugs is caused by the following factors: 1) inadequate drug penetration into tissues, including overcoming BBB [46, 76]; 2) inadequate inhibition of the target [77]; 3) insufficient suppression of the signal cascade [33]; 4) tumor heterogeneity [78-80], and compensatory activation of other pathogenetic mechanisms [81].

It is a challenge to get over these complexities. For example, the problem of glioblastoma heterogeneity consisting in growth of a subpopulation of non-molecular target cells [82] can be solved by prescribing several drugs simultaneously. However, in this case, the risks increase significantly associated with drug interactions and increased toxicity of drug therapy [83].

## Application of patient-derived tumor models

### Patient-derived tumor models

Tumor models, which are created by culturing tumor cells isolated directly from biopsy material of glioblastoma patients, are important tools for studying tumor biology and are widely used in basic neuro-oncological studies. Another area of application of patient-derived models is the selection of effective therapy based on testing different treatment protocols [84-86]. The greatest importance is given here to the direct selection of the most effective therapy for a particular patient. For this purpose, isolated glioblastoma cells are treated with drugs (or their combinations), radiation therapy, or experimental methods of treatment are used.

The main problem of using tumor models to select personalized therapy is that the model inaccurately reproduces the tumor individual biological behavior in the patient’s body [87]. The need to increase the precision of the model to accurately predict the response to the ongoing therapy is an important milestone of translational neuro-oncology [88].

There are three main groups among patient-derived models: 1) *in vitro* models; 2) *in vivo* models; 3) organoid models. Certain types of models have advantages and disadvantages depending on the purpose of their use (see the Table).

**Table:** Brief characterization of main types of patient-derived tumor models

Characteristics	Cell (*in vivo*) cultures	Xenogenic (*in vitro*) models	Organoid models
Difficulty of creation	Low	High	High
Low cost	Yes	No	No
Cost-effectiveness	High	Low	Low
Time expenditure	Low	High	High
Preservation of original tumor biological features	No	Yes	Yes
Modeling of tumor microenvironment	No	Yes	Yes
Allows to assess immune response	No	No	Yes
Allows to rapidly test drugs	Yes	No	Yes

Traditional two-dimensional (2D) monolayer cell cultures found wide application in solving problems of drug testing, primarily due to the ease of creation, high cost-effectiveness, and the possibility to test drugs at high speeds [84, 89]. However, these models have significant limitations in the accuracy of replicating the original tumor due to the formation of monoclonal cell populations, high degree of genetic and morphological homogeneity, absence of intercellular interactions and formation of tumor microenvironment [86, 89–91].

Most genetic and morphological differences between the model and the original tumor material were overcome at *in vivo* orthotopic models in immunodeficient mice. Along with histological features of glioblastoma like infiltrative growth, microvascular proliferation and areas of necrosis presence, orthotopic models are characterized by the presence of other key mutations: TERT, EGFR, PTEN, TP53, BRAF, and IDH1 [92]. The application of these models is significantly limited by the impossibility to study the interaction between the host immune system and the tumor. In addition, widespread implementation of these models is hindered by high cost along with complexity and duration of their cultivation involving specialists in animals with microsurgical manipulation skills [86, 91, 93]. Therefore, *in vivo* models are of interest for basic research of gliomas rather than for patient treatment selection.

The greatest interest in the application of tumor models for personalized therapy is *in vitro* cultivation of three-dimensional models, the so-called organoids. Tumor organoids based on the material obtained from glioblastoma patients sufficiently reflect genotypic and phenotypic features of the original tumor, inter-tumor heterogeneity, ensure preservation of interaction between tumor cells and tumor microenvironment cells, and require less resources and time as compared to existing xenogeneic models of immunodeficient mice [84, 86]. Thus, the presence of major signaling cascades of tumor progression like EGFR expression and its characteristic intratumor heterogeneity in the case of glioblastoma was established for organoids [94]. The presence of immune system cells, i.e. T-killer and macrophages, was also established in organoids [95, 96]. However, the degree of compliance of immune interactions within the model in a patient with glioblastoma is apparently insufficient to reflect all features of the immune system functions [96, 97].

Other significant limitations of organoids are the difficulty of modeling IDH-mutant astrocytomas [96], reproducing the mechanisms of malignant transformation and the ability of tumor migration along tumor vessels [86, 98].

### Application of organoids for personalized therapy in neuro-oncology

Organoids can be used to assess the expression of various molecular targets and to test appropriate drugs in predicting the response to treatment in patients [99]. A number of studies showed that the results of drug efficacy screening on patient-derived organoids correlate with the response to treatment in a patient [96, 100]. Thus, glioblastoma organoids were found to be sensitive to gefitinib in EGFR mutation presence; to trametinib, in tumors with NF1 mutation, to everolimus, in tumors with PI3K mutation [96]. Moreover, comparative studies on results of drug therapy on organoids and clinical outcomes demonstrated that organoids are characterized by appropriate drug resistance even in the presence of target mutations [87, 96]. Thus, the use of tumor models for therapy selection can be considered a more accurate method in terms of predicting tumor response to treatment compared to specific molecular targets identification [87, 96].

A recently published study by Loong et al. [101] presented an attempt to test a fundamentally new concept consisting in sequential determination of the fullest possible genetic and epigenetic profiles of glioblastoma followed by drugs testing in a patient-derived model. The result was the prescription of everolimus, which is not part of standard care, but showed good results in this patient.

An important aspect of the use of patient-derived models is time expenditure. For example, most drug therapy efficacy screening protocols are designed for a period of 1–4 weeks [100]. A number of authors [86, 96, 102] believe that this time frame is optimal for patients with aggressive forms of gliomas as they need to recover from tumor resection and undergo a standard radiation protocol in combination with temodal.

## Conclusion

Despite a large number of translational studies in neuro-oncology, the treatment of brain gliomas remained for many years the most limited in terms of treatment options and is usually limited to the prescription of standard chemoradiotherapy combined with temodal. Attempts to use targeted therapy, which demonstrated significant results in the treatment of many forms of cancer, showed to be ineffective in patients with brain astrocytomas. However, the described clinical observations of positive results in individual cases when these drugs are used indicate that further research is needed of the possibilities to use targeted therapy, to find ways to influence the compensatory mechanisms of the tumor, to open the blood–brain barrier and to identify new molecular targets.

A wide use of patient-derived tumor models allowing one-step testing of a wide range of antitumor drugs will significantly expand the possibilities of individual therapy of gliomas. Currently, we are searching for optimal protocols allowing to create tumor models that genotypically and phenotypically match the patient’s tumor with drug screening results as close as possible to the observed clinical outcomes.

The introduction of individually tailored targeted therapies based on broad tumor profiling and the use of patient-derived tumor models will provide a significant increase in patient life expectancy and reduce treatment costs.

## References

[1] Ostrom Q.T., Cioffi G., Gittleman H., Patil N., Waite K., Kruchko C., Barnholtz-Sloan J.S. (2019). CBTRUS statistical report: primary brain and other central nervous system tumors diagnosed in the United States in 2012–2016.. Neuro Oncol.

[2] (2022). Zlokachestvennye novoobrazovaniya v Rossii v 2021 godu (zabolevaemost’ i smertnost’).

[3] Absalyamova O.V., Kobyakov G.L., Ryzhova M.V., Poddubskiy A.A., Inozemtseva M.V., Lodygina K.S. (2016). Outcomes of application of modern first-line chemotherapy regimens in complex treatment of glioblastoma patients.. Voprosy neirokhirurgii imeni N.N. Burdenko.

[4] Johnson D.R., O’Neill B.P. (2011). Glioblastoma survival in the United States before and during the temozolomide era.. J Neurooncol.

[5] Witthayanuwat S., Pesee M., Supaadirek C., Supakalin N., Thamronganantasakul K., Krusun S. (2018). Survival analysis of glioblastoma multiforme.. Asian Pac J Cancer Prev.

[6] Field K.M., Simes J., Nowak A.K., Cher L., Wheeler H., Hovey E.J., Brown C.S., Barnes E.H., Sawkins K., Livingstone A., Freilich R., Phal P.M., Fitt G. (2015). CABARET/ COGNO investigators; Rosenthal M.A. Randomized phase 2 study of carboplatin and bevacizumab in recurrent glioblastoma.. Neuro Oncol.

[7] Dirks P.B., Meyer M., Reimand J., Lan X., Head R., Zhu X., Kushida M., Bayani J., Pressey J.C., Lionel A., Clarke I.D., Cusimano M., Squire J., Scherer S., Bernstein M., Woodin M.A., Bader G.D. (2014). Single cell derived clonal analysis of human glioblastoma links functional and genomic heterogeneity.. Neuro Oncol.

[8] Patel A.P., Tirosh I., Trombetta J.J., Shalek A.K., Gillespie S.M., Wakimoto H., Cahill D.P., Nahed B.V., Curry W.T., Martuza R.L., Louis D.N., Rozenblatt-Rosen O., Suvà M.L., Regev A., Bernstein B.E. (2014). Single-cell RNA-seq highlights intratumoral heterogeneity in primary glioblastoma.. Science.

[9] Demuth T., Berens M.E. (2004). Molecular mechanisms of glioma cell migration and invasion.. J Neurooncol.

[10] Lathia J.D., Heddleston J.M., Venere M., Rich J.N. (2011). Deadly teamwork: neural cancer stem cells and the tumor microenvironment.. Cell Stem Cell.

[11] Kobyakov G.L., Absalyamova O.V., Bekyashev A.Kh., Golanov A.V., Izmaylov T.R., Konovalov A.N., Naskhletashvili D.R., Potapov A.A., Ryzhova M.V., Smolin A.V., Trunin Yu.Yu., Ulitin A.Yu., Usachev D.Yu. (2020). Practical guidelines for drug treatment of primary tumors of the central nervous system.. Zlokacestvennye opuholi.

[12] Taylor O.G., Brzozowski J.S., Skelding K.A. (2019). Glioblastoma multiforme: an overview of emerging therapeutic targets.. Front Oncol.

[13] Lima F.R., Kahn S.A., Soletti R.C., Biasoli D., Alves T., da Fonseca A.C., Garcia C., Romão L., Brito J., Holanda-Afonso R., Faria J., Borges H., Moura-Neto V. (2012). Glioblastoma: therapeutic challenges, what lies ahead.. Biochim Biophys Acta.

[14] De Bonis P., Anile C., Pompucci A., Fiorentino A., Balducci M., Chiesa S., Lauriola L., Maira G., Mangiola A. (2013). The influence of surgery on recurrence pattern of glioblastoma.. Clin Neurol Neurosurg.

[15] Rapp M., Baernreuther J., Turowski B., Steiger H.J., Sabel M., Kamp M.A. (2017). Recurrence pattern analysis of primary glioblastoma.. World Neurosurg.

[16] Ulitin A.Yu., Zheludkova O.G., Ivanov P.I., Kobyakov G.L., Matsko M.V., Naskhletashvili D.R., Protsenko S.A., Ryzhova M.V. (2022). Practical guidelines for drug treatment of primary tumors of the central nervous system.. Zlokacestvennye opuholi.

[17] Kesari S. (2011). Understanding glioblastoma tumor biology: the potential to improve current diagnosis and treatments.. Semin Oncol.

[18] Hartmann C., Meyer J., Balss J., Capper D., Mueller W., Christians A., Felsberg J., Wolter M., Mawrin C., Wick W., Weller M., Herold-Mende C., Unterberg A., Jeuken J.W., Wesseling P., Reifenberger G., von Deimling A. (2009). Type and frequency of IDH1 and IDH2 mutations are related to astrocytic and oligodendroglial differentiation and age: a study of 1,010 diffuse gliomas.. Acta Neuropathol.

[19] Hegi M.E., Diserens A.C., Gorlia T., Hamou M.F., de Tribolet N., Weller M., Kros J.M., Hainfellner J.A., Mason W., Mariani L., Bromberg J.E., Hau P., Mirimanoff R.O., Cairncross J.G., Janzer R.C., Stupp R. (2005). MGMT gene silencing and benefit from temozolomide in glioblastoma.. N Engl J Med.

[20] Cairncross G., Wang M., Shaw E., Jenkins R., Brachman D., Buckner J., Fink K., Souhami L., Laperriere N., Curran W., Mehta M. (2013). Phase III trial of chemoradiotherapy for anaplastic oligodendroglioma: long-term results of RTOG 9402.. J Clin Oncol.

[21] van den Bent M.J., Brandes A.A., Taphoorn M.J., Kros J.M., Kouwenhoven M.C., Delattre J.Y., Bernsen H.J., Frenay M., Tijssen C.C., Grisold W., Sipos L., Enting R.H., French P.J., Dinjens W.N., Vecht C.J., Allgeier A., Lacombe D., Gorlia T.,  Hoang-Xuan K. (2013). Adjuvant procarbazine, lomustine, and vincristine chemotherapy in newly diagnosed anaplastic oligodendroglioma: long-term follow-up of EORTC brain tumor group study 26951.. J Clin Oncol.

[22] Belyaev A.Yu., Kobyakov G.L., Shmakov P.N., Telysheva E.N., Strunina Yu.V., Usachev D.Yu. (2022). Role of TERT mutation for treatment prognosis in patients with IDH-negative anaplastic astrocytoma.. Voprosy neirokhirurgii imeni N.N. Burdenko.

[23] Stupp R., Hegi M.E., Mason W.P., van den Bent M.J., Taphoorn M.J., Janzer R.C., Ludwin S.K., Allgeier A., Fisher B., Belanger K., Hau P., Brandes A.A., Gijtenbeek J., Marosi C., Vecht C.J., Mokhtari K., Wesseling P., Villa S., Eisenhauer E., Gorlia T., Weller M., Lacombe D., Cairncross J.G., Mirimanoff R.O. (2009). European Organisation for Research and Treatment of Cancer Brain Tumour and Radiation Oncology Groups; National Cancer Institute of Canada Clinical Trials Group. Effects of radiotherapy with concomitant and adjuvant temozolomide versus radiotherapy alone on survival in glioblastoma in a randomised phase III study: 5-year analysis of the EORTC-NCIC trial.. Lancet Oncol.

[24] (2008). Cancer Genome Atlas Research Network. Comprehensive genomic characterization defines human glioblastoma genes and core pathways.. Nature.

[25] Galbraith K., Snuderl M. (2021). Molecular pathology of gliomas.. Surg Pathol Clin.

[26] Chuang D.F., Lin X. (2019). Targeted therapies for the treatment of glioblastoma in adults.. Curr Oncol Rep.

[27] Morgensztern D., McLeod H.L. (2005). PI3K/Akt/mTOR pathway as a target for cancer therapy.. Anticancer Drugs.

[28] Ceccarelli M., Barthel F.P., Malta T.M., Sabedot T.S., Salama S.R., Murray B.A., Morozova O., Newton Y., Radenbaugh A., Pagnotta S.M., Anjum S., Wang J., Manyam G., Zoppoli P., Ling S., Rao A.A., Grifford M., Cherniack A.D., Zhang H., Poisson L., Carlotti C.G. Jr., Tirapelli D.P., Rao A., Mikkelsen T., Lau C.C., Yung W.K., Rabadan R., Huse J., Brat D.J., Lehman N.L., Barnholtz-Sloan J.S., Zheng S., Hess K., Rao G., Meyerson M., Beroukhim R., Cooper L., Akbani R., Wrensch M., Haussler D., Aldape K.D., Laird P.W., Gutmann D.H. (2016). TCGA Research Network; Noushmehr H., Iavarone A., Verhaak R.G. Molecular profiling reveals biologically discrete subsets and pathways of progression in diffuse glioma.. Cell.

[29] Vivanco I., Robins H.I., Rohle D., Campos C., Grommes C., Nghiemphu P.L., Kubek S., Oldrini B., Chheda M.G., Yannuzzi N., Tao H., Zhu S., Iwanami A., Kuga D., Dang J., Pedraza A., Brennan C.W., Heguy A., Liau L.M., Lieberman F., Yung W.K., Gilbert M.R., Reardon D.A., Drappatz J., Wen P.Y., Lamborn K.R., Chang S.M., Prados M.D., Fine H.A., Horvath S., Wu N., Lassman A.B., DeAngelis L.M., Yong W.H., Kuhn J.G., Mischel P.S., Mehta M.P., Cloughesy T.F., Mellinghoff I.K. (2012). Differential sensitivity of glioma- versus lung cancer-specific EGFR mutations to EGFR kinase inhibitors.. Cancer Discov.

[30] Rich J.N., Reardon D.A., Peery T., Dowell J.M., Quinn J.A., Penne K.L., Wikstrand C.J., Van Duyn L.B., Dancey J.E., McLendon R.E., Kao J.C., Stenzel T.T., Ahmed Rasheed B.K., Tourt-Uhlig S.E., Herndon J.E. II, Vredenburgh J.J., Sampson J.H., Friedman A.H., Bigner D.D., Friedman H.S. (2004). Phase II trial of gefitinib in recurrent glioblastoma.. J Clin Oncol.

[31] Franceschi E., Cavallo G., Lonardi S., Magrini E., Tosoni A., Grosso D., Scopece L., Blatt V., Urbini B., Pession A., Tallini G., Crinò L., Brandes A.A. (2007). Gefitinib in patients with progressive high-grade gliomas: a multicentre phase II study by Gruppo Italiano Cooperativo di Neuro-Oncologia (GICNO).. Br J Cancer.

[32] van den Bent MJ, Brandes AA, Rampling R, Kouwenhoven MC, Kros JM, Carpentier AF (2009). Randomized phase II trial of erlotinib versus temozolomide or carmustine in recurrent glioblastoma: EORTC brain tumor group study 26034.. J Clin Oncol.

[33] Hegi M.E., Diserens A.C., Bady P., Kamoshima Y., Kouwenhoven M.C., Delorenzi M., Lambiv W.L., Hamou M.F., Matter M.S., Koch A., Heppner F.L., Yonekawa Y., Merlo A., Frei K., Mariani L., Hofer S. (2011). Pathway analysis of glioblastoma tissue after preoperative treatment with the EGFR tyrosine kinase inhibitor gefitinib — a phase II trial.. Mol Cancer Ther.

[34] Colclough N., Chen K., Johnström P., Strittmatter N., Yan Y., Wrigley G.L., Schou M., Goodwin R., Varnäs K., Adua S.J., Zhao M., Nguyen D.X., Maglennon G., Barton P., Atkinson J., Zhang L., Janefeldt A., Wilson J., Smith A., Takano A., Arakawa R., Kondrashov M., Malmquist J., Revunov E., Vazquez-Romero A., Moein M.M., Windhorst A.D., Karp N.A., Finlay M.R.V., Ward R.A., Yates J.W.T., Smith P.D., Farde L., Cheng Z., Cross D.A.E. (2021). Preclinical comparison of the blood–brain barrier permeability of osimertinib with other EGFR TKIs.. Clin Cancer Res.

[35] Chen C., Cheng C.D., Wu H., Wang Z.W., Wang L., Jiang Z.R., Wang A.L., Hu C., Dong Y.F., Niu W.X., Qi S., Qi Z.P., Liu J., Wang W.C., Niu C.S., Liu Q.S. (2021). Osimertinib successfully combats EGFR-negative glioblastoma cells by inhibiting the MAPK pathway.. Acta Pharmacol Sin.

[36] Makhlin I., Salinas R.D., Zhang D., Jacob F., Ming G.L., Song H., Saxena D., Dorsey J.F., Nasrallah M.P., Morrissette J.J., Binder Z.A., O’Rourke D.M., Desai A.S., Brem S., Bagley S.J. (2019). Clinical activity of the EGFR tyrosine kinase inhibitor osimertinib in EGFR-mutant glioblastoma.. CNS Oncol.

[37] Abousaud M., Faroqui N.M., Lesser G., Strowd R.E., Ramkissoon S.H., Kwatra M., Houston K.S., Hsu F.C., Carter A., Petro R., DeTroye A.T. (2021). Clinical experience using osimertinib in patients with recurrent malignant gliomas containing EGFR alterations.. J Cancer Sci Clin Ther.

[38] Lin B., Ziebro J., Smithberger E., Skinner K.R., Zhao E., Cloughesy T.F., Binder Z.A., O’Rourke D.M., Nathanson D.A., Furnari F.B., Miller C.R. (2022). EGFR, the Lazarus target for precision oncology in glioblastoma.. Neuro Oncol.

[39] Kwatra M.M. (2017). A rational approach to target the epidermal growth factor receptor in glioblastoma.. Curr Cancer Drug Targets.

[40] Kaur B., Khwaja F.W., Severson E.A., Matheny S.L., Brat D.J., Van Meir E.G. (2005). Hypoxia and the hypoxia-inducible-factor pathway in glioma growth and angiogenesis.. Neuro Oncol.

[41] Kreisl T.N., Kim L., Moore K., Duic P., Royce C., Stroud I., Garren N., Mackey M., Butman J.A., Camphausen K., Park J., Albert P.S., Fine H.A. (2009). Phase II trial of single-agent bevacizumab followed by bevacizumab plus irinotecan at tumor progression in recurrent glioblastoma.. J Clin Oncol.

[42] Friedman H.S., Prados M.D., Wen P.Y., Mikkelsen T., Schiff D., Abrey L.E., Yung W.K., Paleologos N., Nicholas M.K., Jensen R., Vredenburgh J., Huang J., Zheng M., Cloughesy T. (2009). Bevacizumab alone and in combination with irinotecan in recurrent glioblastoma.. J Clin Oncol.

[43] Chinot O.L., Wick W., Mason W., Henriksson R., Saran F., Nishikawa R., Carpentier A.F., Hoang-Xuan K., Kavan P., Cernea D., Brandes A.A., Hilton M., Abrey L., Cloughesy T. (2014). Bevacizumab plus radiotherapy–temozolomide for newly diagnosed glioblastoma.. N Engl J Med.

[44] Nayak L., de Groot J., Wefel J.S., Cloughesy T.F., Lieberman F., Chang S.M., Omuro A., Drappatz J., Batchelor T.T., DeAngelis L.M., Gilbert M.R., Aldape K.D., Yung A.W., Fisher J., Ye X., Chen A., Grossman S., Prados M., Wen P.Y. (2017). Phase I trial of aflibercept (VEGF trap) with radiation therapy and concomitant and adjuvant temozolomide in patients with high-grade gliomas.. J Neurooncol.

[45] Peereboom D.M., Ahluwalia M.S., Ye X., Supko J.G., Hilderbrand S.L., Phuphanich S., Nabors L.B., Rosenfeld M.R., Mikkelsen T., Grossman S.A. (2013). New Approaches to Brain Tumor Therapy Consortium. NABTT 0502: a phase II and pharmacokinetic study of erlotinib and sorafenib for patients with progressive or recurrent glioblastoma multiforme.. Neuro Oncol.

[46] Reardon D.A., Vredenburgh J.J., Desjardins A., Peters K., Gururangan S., Sampson J.H., Marcello J., Herndon J.E. II, McLendon R.E., Janney D., Friedman A.H., Bigner D.D., Friedman H.S. (2011). Effect of CYP3A-inducing anti-epileptics on sorafenib exposure: results of a phase II study of sorafenib plus daily temozolomide in adults with recurrent glioblastoma.. J Neurooncol.

[47] Lwin Z., Gomez-Roca C., Saada-Bouzid E., Yanez E., Longo Muñoz F., Im S.A., Castanon E., Senellart H., Graham D., Voss M., Doherty M., Lopez J., Ghori R., Kubiak P., Jin F., Norwood K., Chung H.C. (2020). LBA41 LEAP-005: phase II study of lenvatinib (len) plus pembrolizumab (pembro) in patients (pts) with previously treated advanced solid tumours.. Ann Oncol.

[48] Lombardi G., De Salvo G.L., Brandes A.A., Eoli M., Rudà R., Faedi M., Lolli I., Pace A., Daniele B., Pasqualetti F., Rizzato S., Bellu L., Pambuku A., Farina M., Magni G., Indraccolo S., Gardiman M.P., Soffietti R., Zagonel V. (2019). Regorafenib compared with lomustine in patients with relapsed glioblastoma (REGOMA): a multicentre, open-label, randomised, controlled, phase 2 trial.. Lancet Oncol.

[49] Zhang H.H., Du X.J., Deng M.L., Zheng L., Yao D.C., Wang Z.Q., Yang Q.Y., Wu S.X. (2022). Apatinib for recurrent/ progressive glioblastoma multiforme: a salvage option.. Front Pharmacol.

[50] Zhu Y., Zhao L., Xu Y., Zhan W., Sun X., Xu X. (2022). Combining apatinib and temozolomide for brainstem glioblastoma: a case report and review of literature.. Ann Palliat Med.

[51] Yao H., Liu J., Zhang C., Shao Y., Li X., Feng M., Wang X., Gan W., Zhou Y., Huang Y. (2021). Clinical study of apatinib plus temozolomide for the treatment of recurrent high-grade gliomas.. J Clin Neurosci.

[52] Ge J., Li C., Xue F., Qi S., Gao Z., Yu C., Zhang J. (2021). Apatinib plus temozolomide: an effective salvage treatment for recurrent glioblastoma.. Front Oncol.

[53] Wang Y., Meng X., Zhou S., Zhu Y., Xu J., Tao R. (2019). Apatinib plus temozolomide for recurrent glioblastoma: an uncontrolled, open-label study.. Onco Targets Ther.

[54] Ding X., Sun J., Fan T., Li B. (2018). A case report of targeted therapy with apatinib in a patient with recurrent high grade glioma.. Medicine (Baltimore).

[55] Zhang H., Chen F., Wang Z., Wu S. (2017). Successful treatment with apatinib for refractory recurrent malignant gliomas: a case series.. Onco Targets Ther.

[56] Wang L., Liang L., Yang T., Qiao Y., Xia Y., Liu L., Li C., Lu P., Jiang X. (2017). A pilot clinical study of apatinib plus irinotecan in patients with recurrent high-grade glioma: clinical trial/ experimental study.. Medicine (Baltimore).

[57] Brennan C.W., Verhaak R.G., McKenna A., Campos B., Noushmehr H., Salama S.R., Zheng S., Chakravarty D., Sanborn J.Z., Berman S.H., Beroukhim R., Bernard B., Wu C.J., Genovese G., Shmulevich I., Barnholtz-Sloan J., Zou L., Vegesna R., Shukla S.A., Ciriello G., Yung W.K., Zhang W., Sougnez C., Mikkelsen T., Aldape K., Bigner D.D., Van Meir E.G., Prados M., Sloan A., Black K.L., Eschbacher J., Finocchiaro G., Friedman W., Andrews D.W., Guha A., Iacocca M., O’Neill B.P., Foltz G., Myers J., Weisenberger D.J., Penny R., Kucherlapati R., Perou C.M., Hayes D.N., Gibbs R., Marra M., Mills G.B., Lander E., Spellman P., Wilson R., Sander C., Weinstein J., Meyerson M., Gabriel S., Laird P.W., Haussler D., Getz G., Chin L. (2013). TCGA Research Network. The somatic genomic landscape of glioblastoma.. Cell.

[58] Pitz M.W., Eisenhauer E.A., MacNeil M.V., Thiessen B., Easaw J.C., Macdonald D.R., Eisenstat D.D., Kakumanu A.S., Salim M., Chalchal H., Squire J., Tsao M.S., Kamel-Reid S., Banerji S., Tu D., Powers J., Hausman D.F., Mason W.P. (2015). Phase II study of PX-866 in recurrent glioblastoma.. Neuro Oncol.

[59] Wen P.Y., Touat M., Alexander B.M., Mellinghoff I.K., Ramkissoon S., McCluskey C.S., Pelton K., Haidar S., Basu S.S., Gaffey S.C., Brown L.E., Martinez-Ledesma J.E., Wu S., Kim J., Wei W., Park M.A., Huse J.T., Kuhn J.G., Rinne M.L., Colman H., Agar N.Y.R., Omuro A.M., DeAngelis L.M., Gilbert M.R., de Groot J.F., Cloughesy T.F., Chi A.S., Roberts T.M., Zhao J.J., Lee E.Q., Nayak L., Heath J.R., Horky L.L., Batchelor T.T., Beroukhim R., Chang S.M., Ligon A.H., Dunn I.F., Koul D., Young G.S., Prados M.D., Reardon D.A., Yung W.K.A., Ligon K.L. (2019). Buparlisib in patients with recurrent glioblastoma harboring phosphatidylinositol 3-kinase pathway activation: an open-label, multicenter, multi-arm, phase II trial.. J Clin Oncol.

[60] Chang S.M., Wen P., Cloughesy T., Greenberg H., Schiff D., Conrad C., Fink K., Robins H.I., De Angelis L., Raizer J., Hess K., Aldape K., Lamborn K.R., Kuhn J., Dancey J., Prados M.D. (2005). North American Brain Tumor Consortium and the National Cancer Institute. Phase II study of CCI-779 in patients with recurrent glioblastoma multiforme.. Invest New Drugs.

[61] Reardon D.A., Desjardins A., Vredenburgh J.J., Gururangan S., Friedman A.H., Herndon J.E. II, Marcello J., Norfleet J.A., McLendon R.E., Sampson J.H., Friedman H.S. (2010). Phase 2 trial of erlotinib plus sirolimus in adults with recurrent glioblastoma.. J Neurooncol.

[62] Ma D.J., Galanis E., Anderson S.K., Schiff D., Kaufmann T.J., Peller P.J., Giannini C., Brown P.D., Uhm J.H., McGraw S., Jaeckle K.A., Flynn P.J., Ligon K.L., Buckner J.C., Sarkaria J.N. (2015). A phase II trial of everolimus, temozolomide, and radiotherapy in patients with newly diagnosed glioblastoma: NCCTG N057K.. Neuro Oncol.

[63] Wen P.Y., de Groot J.F., Battiste J., Goldlust S.A., Garner J.S., Friend J., Simpson J.A., Damek D., Olivero A., Cloughesy T.F. (2022). Paxalisib in patients with newly diagnosed glioblastoma with unmethylated MGMT promoter status: final phase 2 study results.. J Clin Oncol.

[64] Wick W., Gorlia T., Bady P., Platten M., van den Bent M.J., Taphoorn M.J., Steuve J., Brandes A.A., Hamou M.F., Wick A., Kosch M., Weller M., Stupp R., Roth P., Golfinopoulos V., Frenel J.S., Campone M., Ricard D., Marosi C., Villa S., Weyerbrock A., Hopkins K., Homicsko K., Lhermitte B., Pesce G., Hegi M.E. (2016). Phase II study of radiotherapy and temsirolimus versus radiochemotherapy with temozolomide in patients with newly diagnosed glioblastoma without MGMT promoter hypermethylation (EORTC 26082).. Clin Cancer Res.

[65] Hainsworth J.D., Shih K.C., Shepard G.C., Tillinghast G.W., Brinker B.T., Spigel D.R. (2012). Phase II study of concurrent radiation therapy, temozolomide, and bevacizumab followed by bevacizumab/everolimus as first-line treatment for patients with glioblastoma.. Clin Adv Hematol Oncol.

[66] Dougherty M.J., Santi M., Brose M.S., Ma C., Resnick A.C., Sievert A.J., Storm P.B., Biegel J.A. (2010). Activating mutations in BRAF characterize a spectrum of pediatric low-grade gliomas.. Neuro Oncol.

[67] Schindler G., Capper D., Meyer J., Janzarik W., Omran H., Herold-Mende C., Schmieder K., Wesseling P., Mawrin C., Hasselblatt M., Louis D.N., Korshunov A., Pfister S., Hartmann C., Paulus W., Reifenberger G., von Deimling A. (2011). Analysis of BRAF V600E mutation in 1,320 nervous system tumors reveals high mutation frequencies in pleomorphic xanthoastrocytoma, ganglioglioma and extra-cerebellar pilocytic astrocytoma.. Acta Neuropathol.

[68] Ballester L.Y., Fuller G.N., Powell S.Z., Sulman E.P., Patel K.P., Luthra R., Routbort M.J. (2017). Retrospective analysis of molecular and immunohistochemical characterization of 381 primary brain tumors.. J Neuropathol Exp Neurol.

[69] Chi A.S., Batchelor T.T., Yang D., Dias-Santagata D., Borger D.R., Ellisen L.W., Iafrate A.J., Louis D.N. (2013). BRAF V600E mutation identifies a subset of low-grade diffusely infiltrating gliomas in adults.. J Clin Oncol.

[70] Vuong H.G., Altibi A.M.A., Duong U.N.P., Ngo H.T.T., Pham T.Q., Fung K.M., Hassell L. (2018). BRAF mutation is associated with an improved survival in glioma-a systematic review and meta-analysis.. Mol Neurobiol.

[71] Wen P.Y., Stein A., van den Bent M., De Greve J., Wick A., de Vos F.Y.F.L., von Bubnoff N., van Linde M.E., Lai A., Prager G.W., Campone M., Fasolo A., Lopez-Martin J.A., Kim T.M., Mason W.P., Hofheinz R.D., Blay J.Y., Cho D.C., Gazzah A., Pouessel D., Yachnin J., Boran A., Burgess P., Ilankumaran P., Gasal E., Subbiah V. (2022). Dabrafenib plus trametinib in patients with BRAF^V600E^-mutant low-grade and high-grade glioma (ROAR): a multicentre, open-label, single-arm, phase 2, basket trial.. Lancet Oncol.

[72] Chamberlain MC. (2013). Salvage therapy with BRAF inhibitors for recurrent pleomorphic xanthoastrocytoma: a retrospective case series.. J Neurooncol.

[73] Li Y., Yang S., Hao C., Chen J., Li S., Kang Z., Kang X., Zhang H., Li W. (2020). Effect of BRAF/MEK inhibition on epithelioid glioblastoma with BRAF^V600E^ mutation: a case report and review of the literature.. Clin Lab.

[74] Lin Z., Xu H., Yang R., Li Z., Zheng H., Zhang Z., Peng J., Zhang X., Qi S., Liu Y., Huang G. (2022). Effective treatment of a BRAF V600E-mutant epithelioid glioblastoma patient by vemurafenib: a case report.. Anticancer Drugs.

[75] Wen P., Alexander S., Yung-Jue B., van den Bent M., Gazzah A., Dietrich S., de Vos F., van Linde M., Lai A., Chi A., Prager G., Campone M., von Bubnoff N., Fasolo A., Lopez-Martin J., Mookerjee B., Boran A., Burgess P., Rangwala F., Subbiah V. (2018). Rare-09. Efficacy and safety of dabrafenib + trametinib in patients with recurrent/refractory BRAF V600E-mutated high-grade glioma (HGG).. Neuro Oncol.

[76] Cruz Da Silva E., Mercier M.C., Etienne-Selloum N., Dontenwill M., Choulier L. (2021). A systematic review of glioblastoma-targeted therapies in phases II, III, IV clinical trials.. Cancers (Basel).

[77] Lassman A.B., Rossi M.R., Raizer J.J., Abrey L.E., Lieberman F.S., Grefe C.N., Lamborn K., Pao W., Shih A.H., Kuhn J.G., Wilson R., Nowak N.J., Cowell J.K., DeAngelis L.M., Wen P., Gilbert M.R., Chang S., Yung W.A., Prados M., Holland E.C. (2005). Molecular study of malignant gliomas treated with epidermal growth factor receptor inhibitors: tissue analysis from North American Brain Tumor Consortium trials 01-03 and 00-01.. Clin Cancer Res.

[78] Sottoriva A., Spiteri I., Piccirillo S.G., Touloumis A., Collins V.P., Marioni J.C., Curtis C., Watts C. (2013). Tavaré S. Intratumor heterogeneity in human glioblastoma reflects cancer evolutionary dynamics.. Proc Natl Acad Sci US A.

[79] Wang X., Prager B.C., Wu Q., Kim L.J.Y., Gimple R.C., Shi Y., Yang K., Morton A.R., Zhou W., Zhu Z., Obara E.A.A., Miller T.E., Song A., Lai S., Hubert C.G., Jin X., Huang Z., Fang X., Dixit D., Tao W., Zhai K., Chen C., Dong Z., Zhang G., Dombrowski S.M., Hamerlik P., Mack S.C., Bao S., Rich J.N. (2018). Reciprocal signaling between glioblastoma stem cells and differentiated tumor cells promotes malignant progression.. Cell Stem Cell.

[80] Uneda A., Kurozumi K., Fujimura A., Fujii K., Ishida J., Shimazu Y., Otani Y., Tomita Y., Hattori Y., Matsumoto Y., Tsuboi N., Makino K., Hirano S., Kamiya A., Date I. (2021). Differentiated glioblastoma cells accelerate tumor progression by shaping the tumor microenvironment via CCN1-mediated macrophage infiltration.. Acta Neuropathol Commun.

[81] Jun H.J., Acquaviva J., Chi D., Lessard J., Zhu H., Woolfenden S., Bronson R.T., Pfannl R., White F., Housman D.E., Iyer L., Whittaker C.A., Boskovitz A., Raval A., Charest A. (2012). Acquired MET expression confers resistance to EGFR inhibition in a mouse model of glioblastoma multiforme.. Oncogene.

[82] Meyer M., Reimand J., Lan X., Head R., Zhu X., Kushida M., Bayani J., Pressey J.C., Lionel A.C., Clarke I.D., Cusimano M., Squire J.A., Scherer S.W., Bernstein M., Woodin M.A., Bader G.D., Dirks P.B. (2015). Single cell-derived clonal analysis of human glioblastoma links functional and genomic heterogeneity.. Proc Natl Acad Sci U S A.

[83] Wen P.Y., Chang S.M., Lamborn K.R., Kuhn J.G., Norden A.D., Cloughesy T.F., Robins H.I., Lieberman F.S., Gilbert M.R., Mehta M.P., Drappatz J., Groves M.D., Santagata S., Ligon A.H., Yung W.K., Wright J.J., Dancey J., Aldape K.D., Prados M.D., Ligon K.L. (2014). Phase I/II study of erlotinib and temsirolimus for patients with recurrent malignant gliomas: North American Brain Tumor Consortium trial 04-02.. Neuro Oncol.

[84] Zhang C., Jin M., Zhao J., Chen J., Jin W. (2020). Organoid models of glioblastoma: advances, applications and challenges.. Am J Cancer Res.

[85] da Hora C.C., Schweiger M.W., Wurdinger T., Tannous B.A. (2019). Patient-derived glioma models: from patients to dish to animals.. Cells.

[86] Pernik M.N., Bird C.E., Traylor J.I., Shi D.D., Richardson T.E., McBrayer S.K., Abdullah K.G. (2021). Patient-derived cancer organoids for precision oncology treatment.. J Pers Med.

[87] Golebiewska A., Hau A.C., Oudin A., Stieber D., Yabo Y.A., Baus V., Barthelemy V., Klein E., Bougnaud S., Keunen O., Wantz M., Michelucci A., Neirinckx V., Muller A., Kaoma T., Nazarov P.V., Azuaje F., De Falco A., Flies B., Richart L., Poovathingal S., Arns T., Grzyb K., Mock A., Herold-Mende C., Steino A., Brown D., May P., Miletic H., Malta T.M., Noushmehr H., Kwon Y.J., Jahn W., Klink B., Tanner G., Stead L.F., Mittelbronn M., Skupin A., Hertel F., Bjerkvig R., Niclou S.P. (2020). Patient-derived organoids and orthotopic xenografts of primary and recurrent gliomas represent relevant patient avatars for precision oncology.. Acta Neuropathol.

[88] Aldape K., Brindle K.M., Chesler L., Chopra R., Gajjar A., Gilbert M.R., Gottardo N., Gutmann D.H., Hargrave D., Holland E.C., Jones D.T.W., Joyce J.A., Kearns P., Kieran M.W., Mellinghoff I.K., Merchant M., Pfister S.M., Pollard S.M., Ramaswamy V., Rich J.N., Robinson G.W., Rowitch D.H., Sampson J.H., Taylor M.D., Workman P., Gilbertson R.J. (2019). Challenges to curing primary brain tumours.. Nat Rev Clin Oncol.

[89] Ledur P.F., Onzi G.R., Zong H., Lenz G. (2017). Culture conditions defining glioblastoma cells behavior: what is the impact for novel discoveries?. Oncotarget.

[90] Seidel S., Garvalov B.K., Acker T. (2015). Isolation and culture of primary glioblastoma cells from human tumor specimens.. Methods Mol Biol.

[91] Yoshida G.J. (2020). Applications of patient-derived tumor xenograft models and tumor organoids.. J Hematol Oncol.

[92] Vaubel R.A., Tian S., Remonde D., Schroeder M.A., Mladek A.C., Kitange G.J., Caron A., Kollmeyer T.M., Grove R., Peng S., Carlson B.L., Ma D.J., Sarkar G., Evers L., Decker P.A., Yan H., Dhruv H.D., Berens M.E., Wang Q., Marin B.M., Klee E.W., Califano A., LaChance D.H., Eckel-Passow J.E., Verhaak R.G., Sulman E.P., Burns T.C., Meyer F.B., O’Neill B.P., Tran N.L., Giannini C., Jenkins R.B., Parney I.F., Sarkaria J.N. (2020). Genomic and phenotypic characterization of a broad panel of patient-derived xenografts reflects the diversity of glioblastoma.. Clin Cancer Res.

[93] Kamb A. (2005). What’s wrong with our cancer models?. Nat Rev Drug Discov.

[94] Furnari F.B., Cloughesy T.F., Cavenee W.K., Mischel P.S. (2015). Heterogeneity of epidermal growth factor receptor signalling networks in glioblastoma.. Nat Rev Cancer.

[95] Bagley J.A., Reumann D., Bian S., Lévi-Strauss J., Knoblich J.A. Fused cerebral organoids model interactions between brain regions. (2017). Nat Methods.

[96] Jacob F., Salinas R.D., Zhang D.Y., Nguyen P.T.T., Schnoll J.G., Wong S.Z.H., Thokala R., Sheikh S., Saxena D., Prokop S., Liu D.A., Qian X., Petrov D., Lucas T., Chen H.I., Dorsey J.F., Christian K.M., Binder Z.A., Nasrallah M., Brem S., O’Rourke D.M., Ming G.L., Song H. (2020). A patient-derived glioblastoma organoid model and biobank recapitulates inter-and intra-tumoral heterogeneity.. Cell.

[97] Zhang B., Shen R., Cheng S., Feng L. (2019). Immune microenvironments differ in immune characteristics and outcome of glioblastoma multiforme.. Cancer Med.

[98] Hambardzumyan D., Bergers G. (2015). Glioblastoma: defining tumor niches.. Trends Cancer.

[99] Gomez G.A., Oksdath M., Brown M.P., Ebert L.M. (2019). New approaches to model glioblastoma in vitro using brain organoids: implications for precision oncology.. Transl Cancer Res.

[100] Driehuis E., Kretzschmar K., Clevers H. (2020). Establishment of patient-derived cancer organoids for drug-screening applications.. Nat Protoc.

[101] Loong H.H.F., Wong A.M., Chan D.T.M., Cheung M.S.H., Chow C., Ding X., Chan A.K.Y., Johnston P.A., Lau J.Y.W., Poon W.S., Wong N. (2020). Patient-derived tumor organoid predicts drugs response in glioblastoma: a step forward in personalized cancer therapy?. J Clin Neurosci.

[102] Jacob F., Ming G.L., Song H. (2020). Generation and biobanking of patient-derived glioblastoma organoids and their application in CAR T cell testing.. Nat Protoc.

